# Incidental Finding of a Rare Urachal Pathology: Urachal Mucinous Cystic Tumour of Low Malignant Potential

**DOI:** 10.1155/2016/5764625

**Published:** 2016-01-03

**Authors:** Luke L. Wang, Heath Liddell, Sharman Tan Tanny, Briony Norris, Sree Appu, David Pan

**Affiliations:** Department of Urology, Monash Health, 823-865 Centre Road, Bentleigh East, VIC 3165, Australia

## Abstract

Urachal mucinous cystic tumours are rare pathological findings with only 23 previously reported cases in the literature. We present the case of a 54-year-old man with an incidentally found urachal mucinous cystic tumour laparoscopically excised. With its known potential to cause pseudomyxoma peritonei, complete surgical excision is important. Long-term cystoscopic and radiological surveillance is also required.

## 1. Introduction

Urachal mucinous cystic tumours are a rare finding of excised urachal masses. Only 23 cases have been previously reported in the literature. Despite their presumed low malignant potential, pseudomyxoma peritonei is a potential adverse sequela and long-term surveillance of urachal mucinous cystic tumours is important.

## 2. Case Presentation

A 54-year-old male was referred for investigation of an incidental abdominal mass abutting the dome of the bladder. It was identified initially on abdominal ultrasound as part of an investigation for hip pain. The patient reported no abdominal pain, lower urinary tract symptoms, or haematuria. His renal function was normal with a serum creatinine of 77 *μ*mol/L. He had no significant past medical history or family history.

**Figure 1:**
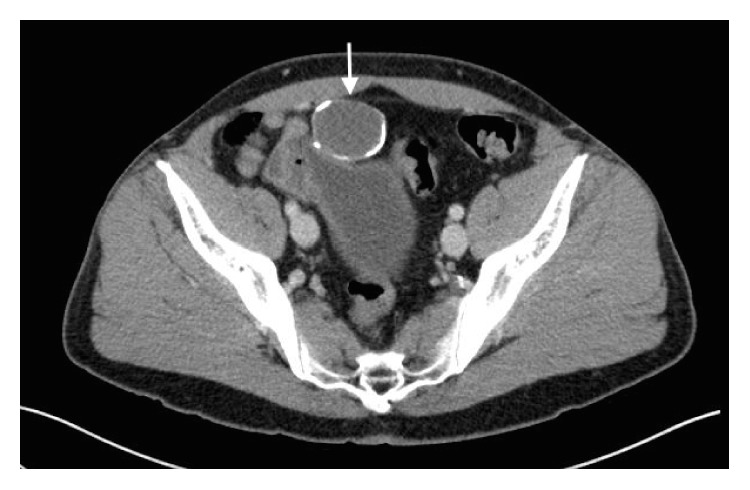
Computed tomography scan showing a cystic lesion anterior to the bladder with calcification within the wall (arrow).

Computed tomography (CT) scan confirmed a cystic lesion measuring 3.5 × 4 cm (Figure 1). It contained internal enhancing solid components and calcification within the wall. Subsequent cystoscopy showed no apparent communication with the bladder. In view of possible urachal malignancy, the patient proceeded to laparoscopic excision of urachal mass, partial cystectomy, and umbilectomy.

**Figure 2:**
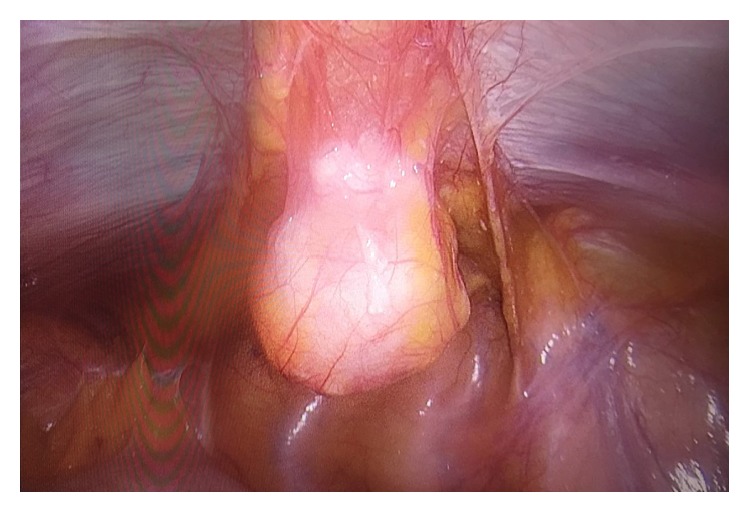
Laparoscopy image showing the mass in the median umbilical ligament superior to the bladder.

Intraoperatively, the mass was cystic in appearance and located 2 cm above the dome of the bladder (Figure 2). The mass was excised with a wide margin, together with a bladder cuff. The umbilicus was then excised en bloc and the mass was removed.

**Figure 3:**
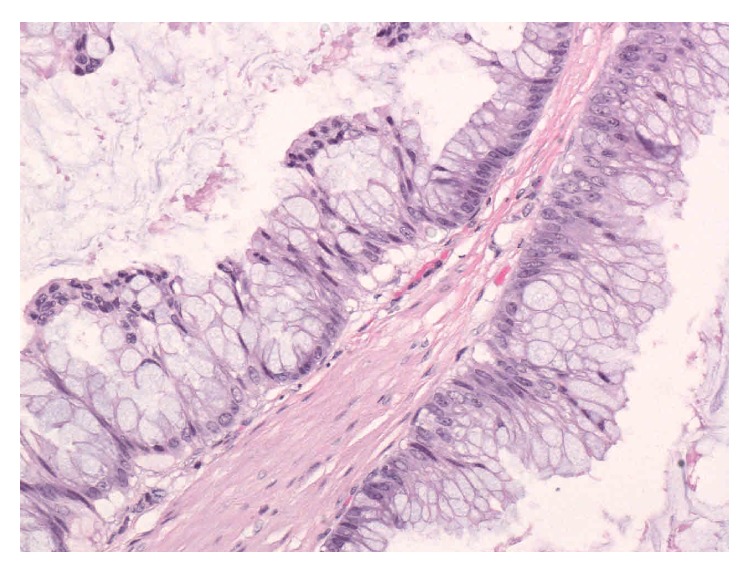
High magnification histopathology image showing goblet cells with pseudostratification and low-grade dysplasia.

Histology revealed the mass to be an urachal mucinous cystic tumour of low malignant potential. Macroscopically, the specimen consisted of umbilical skin with an attached deep fibrous cord that expanded to form an intact unilocular cyst. The other end of the cyst was connected by a segment of fibrofatty tissue to the bladder mucosa. The cyst wall was calcified in areas. No masses were identified within the wall. The cyst had a pasty white lining and contained large amounts of tenacious clear grey mucus. The cyst lumen did not communicate with the umbilicus or bladder segment. Microscopically, the cyst had a calcified fibromuscular wall. The lining was mostly attenuated with few residual strips of flat benign urothelium. Focally, the columnar goblet cell nuclei were slightly enlarged and hyperchromatic with pseudostratification (Figure 3). There was no high-grade dysplasia. The resection margin was clear.

## 3. Discussion

The urachus is an embryological remnant of the allantois. Glandular tumours are the most common neoplasms arising from urachal remnants and they exist on a spectrum from benign mucinous cystic tumours to malignant noncystic neoplasms [1]. The vast majority of reported glandular neoplasms are invasive adenocarcinomas [1]. Rare cases of malignant transformation of urachal adenoma have been reported, with intestinal metaplasia as the most probable underlying mechanism [2].

To our knowledge, a total of 23 cases of urachal mucinous neoplasms of low malignant potential have been reported in the literature [3–5].

A 2014 review of 55 glandular urachal neoplasms included 20 mucinous cystic tumours of low malignant potential [1]. Most commonly, these tumours were incidentally found; however, haematuria, mucusuria, and abdominal pain were also reported. Partial cystectomy with or without urachectomy and umbilectomy was performed in 73% of the cases. Of these 20 cases of mucinous cystic tumour of low malignant potential, 6 were alive without disease at follow-up (range 1–53 months), 1 had died of other causes at 47 months, and no follow-up data were available for the remaining 13 [1].

Pseudomyxoma peritonei is a rare clinical condition defined as extensive intraperitoneal spread of mucus associated with a variety of mucinous tumours, usually arising from the appendix and ovaries [6]. Despite its rarity, pseudomyxoma peritonei has also been reported originating from mucinous urachal neoplasms, including neoplasm of low histologic aggressiveness [3, 6]. Delayed diagnosis or suboptimal management can lead to peritoneal spread and intra-abdominal tumour implants. Therefore, complete surgical excision is paramount.

Long-term cystoscopic and radiological surveillance are required for urachal mucinous cystic tumours. The surveillance cystoscopy protocol for urachal lesions extending to the bladder should follow that for low-grade urothelial carcinoma of the bladder.
